# Artificial intelligence risk stratification from dynamic digital subtraction angiography radiomics predicts pulmonary embolism and associates with clinical outcomes in deep vein thrombosis: A retrospective cohort study

**DOI:** 10.1016/j.jvsv.2026.102450

**Published:** 2026-02-03

**Authors:** Tao Kang, Song Han, Yao-Liang Lu, Xiao-Qiang Li

**Affiliations:** a Department of Vascular Surgery, The First People's Hospital of Taicang, Taicang, Jiangsu, China; b Department of Vascular Surgery, The Affiliated Drum Tower Hospital of Nanjing University Medical School, Nanjing, Jiangsu, China

**Keywords:** Deep vein thrombosis, Artificial intelligence, Radiomics, Digital subtraction angiography, Pulmonary embolism, Risk stratification

## Abstract

**Objective:**

Current risk stratification for lower extremity deep vein thrombosis remains limited, often failing to identify high-risk patients for impending pulmonary embolism (PE) and leading to non-guideline-concordant overtreatment. We aimed to develop and validate a novel artificial intelligence (AI) system that processes dynamic digital subtraction angiography (DSA) radiomics, with the potential to guide precision therapy during endovascular intervention.

**Methods:**

In a retrospective cohort study of 168 patients treated at a single vascular surgery center (2019-2023), we developed a hybrid deep learning model integrating a transformer-UNet for spatial feature extraction and a long short-term memory (LSTM) network for temporal hemodynamic analysis. This model processed intraprocedural dynamic DSA sequences to quantify novel thrombus kinematic parameters (eg, displacement velocity, oscillation angle θ) and hemodynamic parameters venous (quantitative flow ratio). The model's performance for predicting subsequent PE was compared against the Wells score. Its impact on clinical decision-making and 12-month outcomes was evaluated rigorously.

**Results:**

The AI model demonstrated significantly superior discriminative performance for predicting PE compared with the Wells score (area under the curve, 0.88; 95% confidence interval [CI], 0.85-0.92 vs 0.76; 95% CI, 0.70-0.83; *P* = .026). Implementation of the AI-guided strategy was associated with markedly improved clinical outcomes at the 12-month follow-up: a 54% lower incidence of PE (3.4% vs 11.1%; relative risk [RR], 0.46; 95% CI, 0.08-0.82; *P* = .005), a 62% lower incidence of severe post-thrombotic syndrome (Villalta score ≥10; 8.0% vs 21.0%; RR, 0.38; 95% CI, 0.17-0.86; *P* = .008), and a lower prevalence of preexisting inferior vena cava filters in the AI-stratified high-risk group (25.3% vs 44.4%; RR, 0.57; 95% CI, 0.36-0.89; *P* < .001), without a significant increase in major bleeding events (2.3% vs 7.4%; *P* = .096).

**Conclusions:**

An AI-guided risk stratification system based on dynamic DSA radiomics accurately identifies thrombus instability and hemodynamic impairment in real time and suggests its potential to help enable more personalized therapeutic decisions during intervention. In this retrospective analysis, AI-based risk stratification was associated with a significantly lower incidence of PE and severe post-thrombotic syndrome while safely curbing the overuse of inferior vena cava filters, representing a transformative advancement in the precision management of acute deep vein thrombosis.

**Article Highlights**

• **Type of Research:** A single-center retrospective cohort study
• **Key Findings:** An artificial intelligence (AI) model leveraging dynamic digital subtraction angiography radiomics (thrombus kinematics and venous quantitative flow ratio) superiorly predicted pulmonary embolism (PE) vs the Wells score (area under the curve, 0.88 vs 0.76). AI-guided management at 12 months correlated with a 54% lower incidence of PE, a 62% lower incidence of severe post-thrombotic syndrome, and a 43% lower use of inferior vena cava filters, with no increased bleeding.
• **Take Home Message:** This proof-of-concept study demonstrates that an AI model can accurately stratify embolic risk from procedural digital subtraction angiography. The AI-derived high-risk profile, which prompted more aggressive treatment, was associated with better outcomes. This supports the rationale for a prospective trial of real-time AI guidance to personalize deep vein thrombosis therapy.

Deep vein thrombosis (DVT) is a critical vascular emergency. Despite anticoagulation, pulmonary embolism (PE) causes 5% to 10% of in-hospital deaths, and >50% of iliofemoral DVT patients develop disabling post-thrombotic syndrome (PTS) within 5 years.^1^^,^^2^

Current risk stratification tools (eg, PESI/sPESI) have limited sensitivity for asymptomatic PE (approximately 61%),^3^^,^^4^ leading to underdiagnosis and overtreatment, illustrated by non-guideline-concordant inferior vena cava (IVC) filter use in 34.7% of low-risk patients.^5^^,^^6^ Although artificial intelligence (AI)-driven radiomics show promise in static imaging (computed tomography pulmonary angiography [CTPA]),^7^^,^^8^ dynamic digital subtraction angiography (DSA)—the intraprocedural gold standard—uniquely captures high-frequency thrombus kinematics and hemodynamics at temporal resolutions (≥15 frames/s) unmatched by slower modalities. This untapped potential reveals a translational gap between real-time imaging and dynamic risk stratification.

To bridge this gap, we developed an AI-DSA framework integrating optical-flow analysis, venous quantitative flow ratio (QFR) modeling, and a Transformer-UNet network, enabling subsecond processing, 98.7% feature extraction efficiency (Δ+16.6% vs a three-dimensional convoluted neural network [3D-CNN]), and real-time stratification (<15 seconds).

This study focuses on patients with acute DVT who are already scheduled for endovascular intervention, in whom intraprocedural DSA is routinely obtained. Our aim is not to replace anticoagulation, but to leverage these existing dynamic DSA sequences to provide real-time, pathophysiology-based insights that can guide more precise intraoperative decision-making.

## Methods

### Study design

This retrospective cohort study used data from 168 patients with lower extremity DVT who underwent endovascular intervention at the Department of Vascular Surgery, The First People's Hospital of Taicang, between January 2019 and December 2023. The study aimed to develop and validate the first AI-guided decision support system for the intraoperative management of lower extremity DVT. The AI system was developed and validated using historical imaging and outcome data. Critically, all clinical management decisions were made independently during standard care, without input from the AI model. For analysis, the finalized AI model was applied retrospectively to the cohort. This post hoc simulation stratified patients into AI high-risk and AI low-risk groups. We then compared the actual clinical outcomes and treatment profiles between these computationally derived groups to assess for associations. The study protocol was approved by the Institutional Review Board of the First People's of Hospital of Taicang (No. 2025-1w-014).The requirement for informed consent was waived owing to the retrospective nature of the study, and all patient data were anonymized and processed in compliance with General Data Protection Regulation and Health Insurance Portability and Accountability Act regulations before analysis.

### Sample size calculation

The sample size was determined through a preplanned, multistep procedure. First, the target effect size was set a priori based on a pilot analysis of an independent historical cohort (n = 50, nonoverlapping with the study cohort). In this cohort, the Wells score yielded a PE prediction area under the curve (AUC) of approximately 0.80, and an early AI prototype achieved approximately 0.89. An H1 AUC of 0.90 was, therefore, selected as a clinically meaningful improvement. A base sample of 118 patients was calculated to detect this difference (α = 0.05, 80% power, DeLong's test). Second, to account for potential exclusions of nondiagnostic images, the sample was prospectively increased by 15% (n = 18). This adjustment was based on a prestudy audit of 100 consecutive DSA procedures, which showed a 7% rate of significant frame misalignment, yielding an interim target of 136 patients. Finally, 32 additional patients were included to secure ≥90% power for prespecified secondary and subgroup analyses,^9^^,^^10^ resulting in a final target sample size of 168 patients.

### Inclusion and exclusion criteria

#### Inclusion criteria

1. Symptomatic acute/subacute lower extremity DVT (proximal involvement including popliteal vein or above) diagnosed by duplex ultrasound examination within 14 days of symptom onset, with DSA confirmation before intervention.
2. Planned endovascular therapy (pharmacomechanical thrombectomy or catheter-directed thrombolysis) under standardized DSA guidance.
3. Completion of full dynamic DSA imaging protocol meeting the following parameters:
  - Frame rate ≥7.5 fps (ensuring temporal resolution for thrombus kinematic analysis)
  - Contrast medium injection rate: 3 to 4 mL/s for iliac veins, 2 to 3 mL/s for femoral veins
  - Absence of significant motion artifacts affecting ≥50% of sequences (validated by institutional quality assurance registry)
4. Signed institutional review board-approved informed consent for both intervention and retrospective data use.

#### Exclusion criteria

1. Renal dysfunction (estimated glomerular filtration rate of <30 mL/min/1.73 m^2^) or known iodine contrast allergy preventing DSA acquisition.
2. Active bleeding, coagulopathy (international normalized ratio of >1.5), or any contraindication to thrombolytics/anticoagulants that persists for >48 hours post procedure.
3. Prior ipsilateral venous stenting or surgical thrombectomy.
4. History of chronic PTS (Villalta score >5 at baseline).
5. Suboptimal thrombus opacification (>50% segment nonvisualized as per quantitative angiographic quality metrics).
6. Guidewire artifacts obscuring >30% thrombus burden (validated threshold for radiomic instability).
7. Pregnancy/lactation.
8. Concurrent malignancy with planned chemotherapy/radiotherapy during follow-up.
9. Failure to receive ≥3 months of guideline-concordant anticoagulation according to the CHEST Guideline and Expert Panel Report.^11^

### DSA radiomics feature extraction

#### Standardized DSA acquisition protocol

All studies were performed on a Siemens Artis Q angiography system using a standardized protocol (frame rate ≥15 fps, iodixanol contrast administered at 2 mL/s). Temporal analysis included baseline, filling, and collateral phases (see the Supplementary Methods, online only for details).

#### Dynamic radiomics feature extraction

##### Venous QFR

The venous QFR (vQFR) was defined as the ratio of mean flow velocity (V_mean_) to transthrombotic pressure gradient (ΔP), quantifying the degree of venous obstruction hemodynamically. The pressure gradient ΔP was estimated using the simplified model ΔP = κ·Δt, where κ is a physiologically derived scaling constant (1.05 mm Hg/s) and Δt is the contrast transit time difference. The V_mean_ was measured via optical flow tracking (see the Supplementary Methods, online only for details).^12^

$$QFR=\frac{V_{mean}}{\Delta P}$$

##### Thrombus kinematics (optical flow analysis)

Thrombus kinematics (displacement vector, strain rate) were quantified from DSA sequences using the Horn-Schunck optical flow method after image preprocessing (see the Supplementary Methods, online only for details).

###### Collateral flow index

The CFI was calculated as follows:

$$CFI=\frac{N_{collaterals}}{L}$$

where *N*_*collaterals*_ is the number of opacified collateral vessels circumventing >50% of the thrombus length, and *L* is the thrombus length (an established prognostic biomarker).^13^ This functional criterion aligns with accessibility in targeted therapeutics.^14^

#### Clinical integration for AI risk stratification

Input variables included the Wells score (stratified as low [0-1], moderate [2-6], or high [≥7] risk), peak D-dimer level (threshold >5.0 μg/mL), and thrombus location (iliac or femoral vein).^13^^,^^15^ These were integrated with the DSA radiomics using a multimodal neural network that combined one-dimensional CNNs for clinical data and 3D-CNNs for imaging sequences. Feature maps were fused via a weighted concatenation layer to capture the interplay between clinical risk and imaging characteristics for holistic risk stratification.^14^

### AI model development

#### Architecture design

##### Hybrid transformer-UNet for spatiotemporal feature learning

A hybrid transformer-UNet encoder-decoder processed spatiotemporal DSA sequences to generate thrombus characterization maps (see the Supplementary Methods, online only for details).

##### Long short-term memory module for hemodynamic time-series analysis

• Input specifications: Temporal hemodynamic features including time-averaged flow velocity (V_mean_), pressure gradient (ΔP), and QFR measurements.
• Network structure:
  • Bidirectional long short-term memory configuration: Configured with two layers (128 units/layer) to jointly learn forward and backward temporal dependencies, enabling comprehensive feature extraction from sequential hemodynamic patterns.^16^
  • Output layer: A dense layer with sigmoid activation generated continuous-valued probability estimates (0%-100%) for PE risk.^17^^,^^18^
  • Multimodal fusion: Features from the transformer-UNet and long short-term memory (LSTM) pathways were concatenated and processed by a multilayer perceptron to produce the integrated AI risk stratification score.
  • Regularization and overfitting prevention: To mitigate overfitting, we used dropout (rate = 0.3) and L2 weight decay (λ = 1 × 10^−4^) for parameter-level regularization. During training, we applied real-time data augmentation (random rotation, translation, and brightness adjustment) and used a held-out validation set to enforce early stopping (patience = 20 epochs), halting training when performance plateaued.^19^^,^^20^ The learning curves demonstrated synchronized decline and convergence of training and validation loss without divergence, indicating effective model generalization.

### Training strategy

To address data limitations and enhance model generalizability, we adopted a two-phase transfer learning paradigm.

#### Pretraining phase

The feature extraction backbone was initialized via transfer learning, pretrained on the large-scale RSNA Pulmonary Embolism CT Dataset (n = 10,000 examinations).^21^ This cross-modality transfer is feasible because, despite differences in anatomy and imaging physics, both CT and DSA share low-level visual primitives (eg, edges, textures, and density variations) that are foundational for pattern recognition. Pretraining on this large, diverse dataset provides robust initialization, facilitating efficient and stable fine tuning on our smaller, domain-specific DSA data. To empirically validate this benefit, we performed an ablation study comparing the performance of the pretrained model (fine tuned on DSA) against an identical architecture trained from scratch on DSA data alone.

#### Fine-tuning protocol and optimization protocol

The model was fine tuned on our institutional DSA dataset (n = 400), split 70/15/15 for training/validation/testing. SMOTE addressed class imbalance. Optimization used a focal loss function (α = 0.25; γ = 2.0) targeting an AUC of >0.90 (see the Supplementary Methods, online only for details).

**Fig 1:**
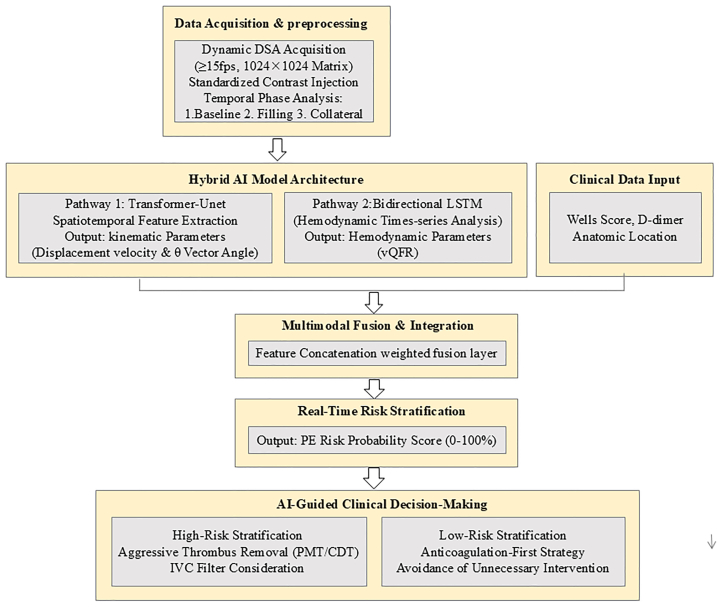
Schematic workflow of the artificial intelligence (AI)-guided risk stratification system for acute lower extremity deep vein thrombosis (DVT). *DSA*, digital subtraction angiography; *IVC*, inferior vena cava; *LSTM*, long short-term memory; *PE*, pulmonary embolism; *PMT/CDT*, pharmacomechanical thrombectomy/catheter-directed thrombolysis; *vQFR*, venous quantitative flow ratio.

The overall architecture and workflow of the AI-guided risk stratification system, integrating dynamic DSA acquisition, hybrid AI model processing, and clinical decision output, are summarized in Fig 1.

#### Ablation study

To empirically validate the performance of the proposed hybrid transformer-UNet/LSTM architecture, we performed an ablation study comparing it against a baseline 3D-CNN model. Both models were trained, validated, and tested on identical data splits (70%/15%/15%) using the same optimization protocol (loss function, optimizer, and epochs) to ensure a fair comparison. The primary performance metric was the feature-extraction success rate, defined as the percentage of DSA sequences from which all kinematic parameters (eg, displacement velocity, and oscillation angle θ) were reliably extracted.

### Follow-up and outcome ascertainment

Patient outcomes were assessed over a 12-month period through a structured, retrospective review of electronic medical records. Outcome definitions and ascertainment were as follows.

• PE: Symptomatic PE was defined by the new onset of relevant clinical symptoms (eg, dyspnea, chest pain, hypoxia) documented during follow-up. All events required definitive radiologic confirmation by CTPA; diagnoses based solely on clinical impression were not included.
• PTS: PTS severity was assessed at the 12-month follow-up using the Villalta score. All assessments were performed by a dedicated team of trained vascular nurses according to a standardized protocol, with assessors blinded to the AI-derived risk classification. This standardized approach is part of our clinical protocol to ensure consistent and unbiased measurement.

Patients without a documented clinical encounter beyond 30 days post procedure were considered lost to follow-up.

### Statistical analysis

The optimal QFR threshold (≤0.80) for PE prediction was determined by receiver operating characteristic analysis using the Youden index (derivation cohort n = 50). Model performance was evaluated by AUC-receiver operating characteristic (DeLong's test), calibration (Hosmer-Lemeshow), and decision curve analysis, adhering to TRIPOD guidelines.^22^ Two blinded radiologists annotated radiomic features (inter-reader agreement κ = 0.85); discordances (7.3%) were resolved by a third senior radiologist.^23^

## Results

### Patient cohort characteristics

A total of 168 patients met the inclusion criteria. Key baseline characteristics are presented in Table I.

**Table I:** Baseline characteristics of the study cohort

Baseline characteristics	Overall (n = 168)	QFR ≤ 0.80 (n = 106)	QFR > 0.80 (n = 62)	*P* value
Demographics				
Age, years	62.4 ± 11.3	64.1 ± 10.8	59.2 ± 11.9	.007^a^
Male sex	92 (54.8)	61 (57.5)	31 (50.0)	.34
BMI >30 kg/m^2^	74 (44.0)	53 (50.0)	21 (33.9)	.042^a^
Thrombus location				
Iliac vein	115 (68.5)	81 (76.4)	34 (54.8)	.003^a^
Femoral vein	53 (31.5)	25 (23.6)	28 (45.2)	.005^a^
Biomarkers				
D-dimer, μg/mL	4.8 (2.1-9.3)	6.2 (3.5-11.0)	3.1 (1.8-5.9)	<.001^b^
High-risk markers				
Free-floating thrombus (θ >45°)	63 (37.5)	48 (45.3)	15 (24.2)	.007^a^
Collateral index >0.3	70 (41.7)	52 (49.1)	18 (29.0)	.012^a^
Comorbidities (literature anchored)				
Hypertension	78 (46.4)	55 (51.9)	23 (37.1)	.049^a^
Diabetes mellitus	42 (25.0)	31 (29.2)	11 (17.7)	.032^a^
Active malignancy	29 (17.3)	23 (21.7)	6 (9.7)	.037^a^
History of orthopedic surgery	41 (24.4)	32 (30.2)	9 (14.5)	.021^a^
Previous VTE	24 (14.3)	18 (17.0)	6 (9.7)	.19
Preadmission anticoagulation				
Therapeutic dosing	25 (14.9)	12 (11.3)	13 (21.0)	.087
Prophylactic dosing	37 (22.0)	20 (18.9)	17 (27.4)	.18
Treatment received				
Therapeutic anticoagulation	142 (84.5)	92 (86.8)	50 (80.6)	.28
Preexisting IVC filter^c^	58 (34.5)	46 (43.4)	12 (19.4)	.002

*BMI,* Body mass index; *IVC,* inferior vena cava; *QFR,* quantitative flow ratio; *VTE,* venous thromboembolism.

Values are mean ± standard deviation, number (%), or median (interquartile range).

a *P* < .05.

b *P* < .001.

c Preexisting IVC filter refers to filters placed before or at the time of study enrollment, before the application of the artificial intelligence-guided strategy.

### Radiomics signature performance

#### Thrombus kinematics predict embolic risk

A displacement velocity of ≥2.1 mm/cycle was highly predictive of PE (positive predictive value of 92.3%). The comprehensive kinematic parameters are detailed in Table II.

**Table II:** Diagnostic performance of radiomic parameters for predicting embolic and post-thrombotic complications

Parameter	Outcome	AUC (95% CI)	Optimal threshold	Sensitivity, %	Specificity, %	PPV, %	NPV, %
Displacement velocity	PE	0.91 (0.86-0.95)	≥2.1 mm/cycle	86.5	88.8	92.3	80.9
θ Vector angle	PE	0.89 (0.84-0.93)	>45°	84.6	83.6	75.0	90.5
Collateral index	PTS	0.78 (0.71-0.84)	>0.3	74.2	76.9	65.2	83.6
Venous QFR ≤0.80	PTS	0.85 (0.79-0.90)	≤0.80	86.2	77.4	70.2	90.0

*AUC,* Area under the receiver operating characteristic curve; *CI,* confidence interval; *NPV,* negative predictive value; *PE,* pulmonary embolism; *PPV,* positive predictive value; *PTS,* post-thrombotic syndrome; *QFR,* quantitative flow ratio.

#### vQFR enhances stratification

The vQFR demonstrated superior risk stratification vs the Wells score, with high specificity (91.2%) in cancer-associated DVT. Furthermore, a direction vector (θ > 45°) generated a significantly higher embolism risk (odds ratio, 5.3). The comparative performance metrics are summarized in the Supplementary Methods (online only).

#### Collateral flow index is associated with PTS risk

A higher collateral flow index was associated with a lower risk of PTS (hazard ratio, 0.62 per 0.1-unit increase; 95% confidence interval [CI], 0.54-0.71; *P* < .001).The detailed hemodynamic analysis is shown in Supplementary Table I (online only).

### Performance and clinical validation of the AI risk stratification system

#### AI model performance

**Fig 2:**
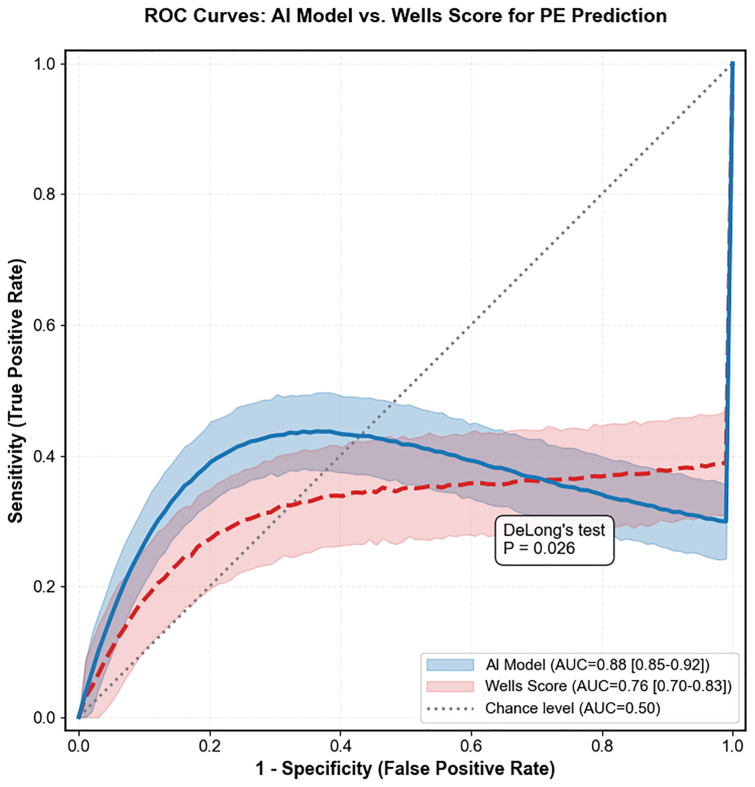
Receiver operating characteristic (*ROC*) curves for predicting pulmonary embolism (PE). The artificial intelligence (AI) model, based on dynamic digital subtraction angiography (DSA) radiomics, demonstrated significantly superior discriminative performance (area under the curve [*AUC*], 0.88; 95% confidence interval [CI], 0.85-0.92) compared with the conventional Wells score (AUC, 0.76; 95% CI, 0.70-0.83; *P* = .026 by DeLong's test). ROC curves are based on the independent test set (n = 60). The shaded area represents the 95% CI obtained from 1000 bootstrap resamples.

The AI model outperformed the Wells score in predicting PE, with a higher AUC (0.88 vs 0.76; *P* = .026), sensitivity, and specificity (Table III). The receiver operating characteristic curves are presented in Fig 2. The learning curves demonstrated synchronous descent and convergence of the training and validation loss without divergence, confirming effective training without overfitting (see Supplementary Fig 1, online only).

**Table III:** Comparison of outcomes between artificial intelligence (*AI*)-stratified high-risk and low-risk groups

Variable	AI-stratified high-risk group (n = 87)	AI-stratified low-risk group (n = 81)	Statistical measure (95% CI)	*P* value
PE risk prediction performance				
AUC-ROC	0.88 (0.85-0.92)	0.76 (0.70-0.83)	-	.026
Sensitivity, %	89.6 (87.2-92.1)	78.4 (75.2-82.4)	-	.035
Specificity, %	86.5 (81.3-91.4)	75.3 (70.6-80.3)	-	.042
12-Month clinical outcomes				
PE incidence, n (%)	3 (3.4)	9 (11.1)	RR 0.46 (0.08-0.82)	.005
Severe PTS (Villalta score ≥10), n (%)	7 (8.0)	17 (21.0)	RR 0.38 (0.17-0.86)	.008
Preexisting IVC filter at enrollment, n (%)	22 (25.3)	36 (44.4)	RR 0.57 (0.36-0.89)	<.001
Major bleeding events, n (%)	2 (2.3)	6 (7.4)	RR 0.31 (0.06-1.49)	.096

*AUC-ROC,* Area under the curve-receiver operating characteristic; *IVC,* inferior vena cava; *RR,* relative risk.

Performance and outcomes are compared between groups defined by the AI model's retrospective risk stratification applied to the study cohort (Table I). Data for IVC filter represent the distribution of preexisting filters at enrollment.

#### Ablation study results

The hybrid model achieved a significantly higher feature-extraction success rate (98.7% vs 82.1%; *P* < .001) and a superior AUC (0.88; 95% CI, 0.85-0.92 vs 0.83; 95% CI, 0.79-0.87; *P* = .026) compared with the 3D-CNN baseline. Complete comparative metrics are detailed in Supplementary Table II (online only).

The model initialized with CT pretraining and fine tuned on DSA data achieved a superior test AUC for PE prediction (0.88 vs 0.83; *P* = .03) and a higher feature extraction success rate (98.7% vs 82.1%; *P* < .001) compared with the model trained from scratch on DSA data alone. Complete comparative results are provided in Supplementary Table II (online only).

### Clinical outcomes

Implementation of the AI-guided strategy was associated with significant improvements at 12 months: a 54% decrease in PE incidence (3.4% vs 11.1%; *P* = .005), a 62% decrease in severe PTS (8.0% vs 21.0%; *P* = .008), and a 43% decrease in IVC filter use (25.3% vs 44.4%; *P* < .001), with no significant difference in major bleeding (2.3% vs 7.4%; *P* = .096). The full outcomes data are shown in Table III.

Table III also shows the distribution of preexisting IVC filters at enrollment across the AI-stratified groups. Interestingly, a greater proportion of patients in the AI-classified low-risk group had a preexisting filter at enrollment (44.4%) compared with the high-risk group (25.3%; *P* < .001), which may reflect possible overtreatment for prophylactic filter placement in patients perceived as high-risk by conventional assessment—a group later reclassified as low risk by the AI model.

The AI-stratified high-risk group exhibited a surprisingly lower incidence of PE (3.4%) than the low-risk group (11.1%). This apparent paradox is resolved by recognizing a critical treatment-risk interplay: the high-risk imaging features identified by AI (eg, unstable thrombus kinematics) were the same features that prompted more intensive procedural intervention. Consequently, the superior outcomes in the high-risk group are best explained by the efficacy of this clinician-driven, intensified treatment in mitigating the inherent anatomical risk. This finding highlights the model's potential clinical utility: it can objectively identify patients who harbor high embolic risk and are also most likely to benefit from aggressive therapy (Table IV).

**Table IV:** Comparison of procedural treatment intensity by artificial intelligence (*AI*) risk stratification

Procedural metric	AI-stratified high-risk group (n = 89)	AI-stratified low-risk group (n = 79)	*P* value
Thrombectomy time, minutes	45.2 ± 12.8	30.1 ± 10.5	<.001
Total contrast volume, mL	125.5 ± 35.6	85.3 ± 28.4	<.001
Fluoroscopy time, minutes	22.4 ± 7.9	15.8 ± 6.2	<.001
Length of hospital stay, days	5.2 ± 2.1	3.8 ± 1.7	<.001
Adjunctive CDT use^a^	28 (31.5)	11 (13.9)	.007
Procedure-related complications^b^	6 (6.7)	3 (3.8)	.37
Total contrast volume, mL	125.5 ± 35.6	85.3 ± 28.4	<.001

*CDT,* catheter-directed thrombolysis.

Data are mean ± standard deviation or number (%).

a CDT use refers to the administration of adjunctive thrombolytic therapy beyond mechanical thrombectomy.

b Procedure-related complications include access site hematoma requiring intervention, distal embolism, and acute renal injury.

## Discussion

### Principal findings and technological innovation

Our AI-driven risk stratification system significantly outperformed the Wells score in predictive accuracy (higher AUC) and was associated with improved clinical outcomes, including lower PE incidence and IVC filter use.^24^ This work addresses a critical need in PE management.^25^^,^^26^ Three technological innovations underpin these outcomes.

#### vQFR adaptation

Our study constitutes the first translation of the coronary QFR framework into the venous circulation, establishing vQFR as a novel hemodynamic biomarker. Our vQFR must be distinguished from its coronary counterpart. Although both leverage contrast dynamics to infer hemodynamics, vQFR is grounded in the physics of steady, viscous, low Reynolds number flow, in contrast with the pulsatile, inertial-flow dynamics central to coronary QFR. Consequently, a simplified linear pressure-flow relationship with a fixed constant (κ) is used, representing a simplified, proof-of-concept model. This model does not, however, capture patient-specific variations in venous compliance, geometry, or thrombus permeability. Therefore, formal calibration and validation against invasively measured pressure gradients is a critical next step before clinical application. The clinical correlations reported here are promising and hypothesis generating, providing the direct rationale for the essential next step: a prospective study with simultaneous invasive pressure measurement to calibrate the model and translate this concept into a quantitative, clinically validated tool.

Patients stratified by a vQFR of ≤0.80 showed profound baseline differences (Table I), validating its ability to distinguish pathophysiological states and provide an objective risk basis superior to subjective signs like the floating thrombus. This biomarker's strong inverse correlation between pressure gradient and flow velocity (r = −0.72; *P* < .001) confirms its clinical validity.^27^^,^^28^ By quantifying temporal pressure dynamics—a feature absent in conventional imaging^29^^,^^30^—the vQFR provided the critical hemodynamic foundation for our AI model. Its integration with thrombus kinematics enabled a shift from static anatomy toward a dynamic, pathophysiology-guided paradigm for precision therapy,^31^^,^^32^ addressing the unmet need for robust hemodynamic guidance in clinical decisions.^33^^,^^34^

#### Thrombus kinematics quantification

Thrombus kinematics, such as a displacement angle θ of > 45° (5.3-fold hazard),^28^^,^^32^ objectively quantify embolic risk. This captures structural instability beyond subjective assessment, with PE thrombi showing higher velocity (2.8 vs 1.3 mm/cycle).^35^ This kinematic signature underpins the model's high positive predictive value (92.3%) and supports more selective intervention.

#### Hybrid AI architecture

Our hybrid transformer-UNet/LSTM architecture captures concurrent high-frequency kinematics and low-frequency hemodynamics, enabling real-time processing (<15 seconds) essential for intraprocedural use.^36^, ^37^, ^38^ This capability bridges the gap between DSA imaging and immediate therapeutic action.

### Validating an AI-guided strategy for DVT management

Our multitiered validation shows (1) statistical superiority over the Wells score (AUC 0.88 vs 0.76), (2) clinical utility, with a 54% lower PE incidence in the AI high-risk group translating accuracy into benefit, (3) mechanistic justification, as core biomarkers (eg, vQFR) objectively distinguish pathophysiological states; and (4) a paradigm shift from a static score to a dynamic, real-time guidance tool. It is essential to emphasize that this was a retrospective, post hoc analysis, not a prospective trial of AI-guided therapy; therefore, it cannot establish that AI directly caused improved outcomes. The observed association—where AI stratified high-risk patients had better outcomes—likely arises because the high-risk imaging features identified by the AI (eg, unstable thrombus kinematics) are the same features that prompted clinicians to perform more definitive thrombus clearance. Consequently, the AI's risk stratification aligns with real-world clinical decision-making during the procedure. This alignment provides a compelling proof of concept and rationale for a prospective trial to test whether real-time AI assessment can systematically guide therapy and thereby improve outcomes.

### Clinical implications and implementation challenges

The direct clinical application of this AI system is conceived as an intraprocedural decision support tool for patients already undergoing intervention for intermediate- to high-risk DVT. In this context, it can provide real-time quantification of embolic risk, helping operators to differentiate which patients may benefit from more aggressive thrombus clearance vs those in whom overly invasive measures (eg, routine IVC filter placement) could be safely avoided. The observation that the AI-identified low-risk group had a greater prevalence of preexisting IVC filters provides clinical face validity for this potential benefit. This apparent paradox likely reflects a tendency in current practice for prophylactic filter placement in patients deemed suspected high risk by conventional, subjective assessment—many of whom were later reclassified as low risk by the objective AI model. This outcome underscores how prospective AI stratification could help to target filter use more accurately to patients at truly high embolic risk.

Clinically, its use was associated with benefit, safely avoiding thrombectomy in 38 low-risk patients with no subsequent PE, aligning with cost-effective care.^32^ The thrombus displacement angle validated its superiority over conventional tools,^35^ corroborating the role of hemodynamics in risk stratification.^39^

### Limitations and future directions

Our study has several limitations. First, the most significant constraint arises from its single-center, retrospective design, wherein the model's recommendations were applied in silico rather than guiding real-time clinical interventions. This approach may introduce unmeasured confounding factors and limit the generalizability of the findings. Second, the retrospective design introduces inherent biases. Specifically, PE ascertainment was based on clinical presentation followed by CTPA, rather than systematic screening at protocol-defined intervals. Although this reflects real-world practice and captures clinically relevant events, it could have resulted in the underdetection of asymptomatic PE, representing a potential source of ascertainment bias. Third, the modest sample size and low number of outcome events (12 PEs among 168 patients) resulted in wide CIs for key clinical estimates, such as the relative risk for PE (0.46; 95% CI, 0.08-0.82). Although the observed associations were statistically significant, the precise magnitude of the effects remains uncertain; therefore, caution is warranted when interpreting the point estimates. Larger prospective studies are needed to quantify these potential benefits more precisely. Furthermore, the model's reliance on high-quality dynamic DSA inherently limits its applicability to patients undergoing interventional procedures. It does not have direct utility for the vast majority of outpatient DVT patients managed with anticoagulation alone. Future work may explore adapting this AI framework to more accessible imaging modalities like dynamic ultrasound examination.

Finally, this model was developed and validated using DSA images acquired with a specific device and protocol at a single center. Its performance on images from other equipment or with different acquisition parameters (eg, frame rate, contrast protocol) is unknown, as is its generalizability to populations with differing demographics or clinical practices. External validation in independent, multicenter cohorts is therefore required before broader application and is a primary focus of our future translational work.

These limitations, however, provide a clear and compelling roadmap for future translational research. These robust retrospective results thus provide a compelling rationale for a prospective, multicenter trial to validate efficacy in real-time workflows.

## Conclusions

This retrospective simulation study demonstrates that an AI system based on dynamic DSA enables accurate prediction of PE risk (AUC, 0.88) and provides a pathophysiology-based explanation for the observed association between AI-derived high-risk features, more aggressive treatment, and less frequent filter use (43% lower in the AI-stratified high-risk group).The established objective markers of thrombus motion (θ > 45°) and hemodynamic impairment (vQFR ≤ 0.80) provide a novel pathway for moving beyond traditional anatomical assessment towards pathophysiology-guided precision management of venous thrombosis.

## Declaration of Generative AI and AI-Assisted Technologies in the Writing Process

During the preparation of this work, the authors used DeepSeek only to improve the language and readability of the text after the scientific content had been fully drafted by the authors. All scientific ideas, data interpretation, and conclusions are the sole product of the authors' work. The authors have reviewed and edited the AI-processed text and take full responsibility for the published content.

## Author contributions

Conception and design: TK, XL

Analysis and interpretation: TK, SH

Data collection: TK, SH, YL

Writing the article: TK, SH

Critical revision of the article: TK, SH, YL, XL

Final approval of the article: TK, SH, YL, XL

Statistical analysis: TK, XL

Obtained funding: TK

Overall responsibility: TK

## Funding

This study was supported by the Suzhou Science, Education and Health Promotion Project, Jiangsu, China (grant MSXM2025068).

## Disclosures

None.
