# Supplementary material for: Artificial intelligence risk stratification from dynamic digital subtraction angiography radiomics predicts pulmonary embolism and associates with clinical outcomes in deep vein thrombosis: A retrospective cohort study
Source: J Vasc Surg Venous Lymphat Disord. 2026 Feb 3;14(3):102450. doi: 10.1016/j.jvsv.2026.102450 (PMC12954298; doi:10.1016/j.jvsv.2026.102450)
Supplement: Supplementary Material [file mmc1.docx]

1. **Detailed DSA Acquisition Protocol**​

All studies were performed using a standardized protocol on a Siemens Artis Q angiography system. Key acquisition parameters were as follows: a frame rate ≥15 fps, a field of view of 40 × 40 cm with consistent geometry to minimize distortion, and a contrast protocol administering Iodixanol (320 mgI/mL) at 2 mL/s via dorsalis pedis access (total volume of 60 mL). Temporal analysis was segmented into three phases:

1. **Baseline Phase (10 sec pre-contrast):​**​ Assessed inherent thrombus mobility under respiratory gating, where a displacement (Δs) ≥1.5 mm defined a mobile thrombus.
2. **Filling Phase (0-5 sec post-contrast):​**​ Analyzed proximal hemodynamics, with mean flow velocity (V_mean_) quantified using optical flow tracking.
3. **Collateral Phase (5-10 sec post-contrast):​**​ Assessed venous drainage patterns, where the collateral index (T_collateral_/T_main_) >0.3 defined impaired drainage ^1^.

​**2. Hemodynamic Parameter Estimation and Key Findings**​

Thrombus kinematics and hemodynamic parameters were quantified from dynamic DSA sequences preprocessed with Gaussian filtering, using the robust Horn-Schunck optical flow method for parameter extraction ^2^. Displacement Vector (mm/s) was calibrated to physical scale using magnification factors and vessel markers ^3^, and Strain Rate (%/s) was derived to quantify shear-induced changes at the thrombus-endothelium interface ^4^.

1. **Optical Flow Kinetic Parameters:​**​ Thrombus displacement velocity during the baseline phase proved to be a significant discriminator. The mean displacement was markedly higher in patients who developed pulmonary embolism (PE) compared to those who did not (2.8 ± 0.9 mm/ cardiac cycle vs. 1.3 ± 0.6 mm/cardiac cycle, respectively; Cohen’s d = 1.87, p < 0.001). A direction vector angle >45°—a potential intervention threshold—was present in 84.6% (44/52) of the PE(+) group versus 16.4% (19/116) of the PE(-) group (Odds Ratio [OR] = 5.3, p < 0.001).
2. **Collateral Index:​**​ Assessment during the collateral phase revealed substantially impaired drainage in the PE(+) group (0.41 ± 0.12 vs. 0.19 ± 0.08; Cohen’s d = 2.25, p < 0.001), establishing it as a robust predictor for post-thrombotic syndrome (PTS).
3. **Venous Quantitative Flow Ratio (vQFR):​**​ The trans‑thrombotic pressure gradient (ΔP) was estimated using the simplified linear model ΔP = κ·Δt, where Δt is the contrast transit time difference derived from DSA ^5^. ​This linear formulation is physiologically justified for the venous system, where low‑velocity, low‑Reynolds‑number flow is dominated by viscous forces, rendering the pressure‑flow relationship more amenable to linear simplification than in pulsatile arteries. The scaling constant κ (1.05 mmHg/s) was derived from fundamental fluid‑dynamics principles under the assumptions of steady, laminar venous flow and fixed segment geometry, and represents a ​physiologically plausible scaling factor​ informed by characteristic venous dimensions and established pressure ranges in the literature ^6^. V_mean_(cm/s) was measured using subpixel optical flow tracking of the contrast front, averaged over three cardiac cycles ^7^. The ΔP was significantly elevated in the PE(+) group (18.3 ± 4.2 mmHg vs. 6.7 ± 2.1 mmHg; r = -0.72, p < 0.001). A vQFR value ≤ 0.80 was a powerful differentiator, present in 92.3% (48/52) of PE(+) patients compared to 50.0% (58/116) of PE(-) patients (OR = 12.4, p < 0.001), demonstrating high specificity (91.2%) in the cancer subgroup.

​**3. Hybrid Transformer-UNet Architecture and Processing**​

A hybrid Transformer-UNet encoder-decoder processed spatiotemporal DSA sequences to generate thrombus characterization maps (location, volume, organization) ^8^. The encoder was a 4-stage Vision Transformer (ViT) with a patch size of 16×16 ^9, 10^, and the decoder was a 3D-UNet with skip connections for effective spatiotemporal feature fusion ^11^. The input consisted of dynamic DSA sequences (3 phases × 15 fps × 1024×1024 matrix) ^12^.

​**4. Model Fine-Tuning and Optimization Protocol**​

The model was fine-tuned on our institutional dynamic DSA dataset (2018–2023; n=400 patients, 16,500 frames). The dataset was partitioned into 70% for training (n=280 patients), 15% for validation (n=60; used for early stopping and hyperparameter monitoring), and 15% for held-out testing (n=60). Given the imbalanced distribution of outcomes, the SMOTE algorithm was employed to create a balanced training set with a DVT-positive to negative ratio of 1:3 ^13^. The model was optimized by minimizing a multi-task focal loss function (α=0.25, γ=2.0) ^14^, with performance targets set at an AUC >0.90 (95% CI: 0.88–0.93), sensitivity >90%, and specificity >80% for the primary outcome.

1. He G, Ling R, Wei L, Lu H, Gu Y, Zhu Y. CTP-defined collaterals is a better predictor of intracranial atherosclerotic stenosis-related large-vessel occlusion than multiphase CTA-defined collaterals. J Cereb Blood Flow Metab. 2025;45(8):1569-80.

2. Adiv G. Determining three-dimensional motion and structure from optical flow generated by several moving objects. IEEE Trans Pattern Anal Mach Intell. 1985;7(4):384-401.

3. Kim KA, Choi SY, Kim R. Endovascular Treatment for Lower Extremity Deep Vein Thrombosis: An Overview. Korean J Radiol. 2021;22(6):931-43.

4. Yao M, Ma J, Wu D, Fang C, Wang Z, Guo T, et al. Neutrophil extracellular traps mediate deep vein thrombosis: from mechanism to therapy. Front Immunol. 2023;14:1198952.

5. Elbasha K, Alotaibi S, Samy M, Mankerious N, Toelg R, Geist V, et al. Quantitative flow ratio of the donor coronary artery supplying a chronic total occlusion territory. Clin Res Cardiol. 2025;114(8):1041-8.

6. Meissner MH, Moneta G, Burnand K, Gloviczki P, Lohr JM, Lurie F, et al. The hemodynamics and diagnosis of venous disease. J Vasc Surg. 2007;46 Suppl S:4S-24S.

7. Zhao L, Chen BH, Tang H, Wang YY, Gu ZY, An DA, et al. The association between cardiac T2*BOLD and quantitative flow ratio (QFR) in the diagnosis of stenotic coronary arteries in patients with multi-vessel coronary artery disease. Radiol Med. 2024;129(8):1184-96.

8. Ma Y, Guo Y, Cui W, Liu J, Li Y, Wang Y, et al. SG-Transunet: A segmentation-guided Transformer U-Net model for KRAS gene mutation status identification in colorectal cancer. Comput Biol Med. 2024;173:108293.

9. Mohammadi S, Ahmadi Livani M. Enhanced breast mass segmentation in mammograms using a hybrid transformer UNet model. Comput Biol Med. 2025;184:109432.

10. Yan Y, Liu R, Chen H, Zhang L, Zhang Q. CCT-Unet: A U-Shaped Network Based on Convolution Coupled Transformer for Segmentation of Peripheral and Transition Zones in Prostate MRI. IEEE J Biomed Health Inform. 2023;27(9):4341-51.

11. Zhang Y, Chung ACS. Retinal Vessel Segmentation by a Transformer-U-Net Hybrid Model With Dual-Path Decoder. IEEE J Biomed Health Inform. 2024;28(9):5347-59.

12. Deng Z, Zhang W, Chen K, Zhou Y, Tian J, Quan G, et al. TT U-Net: Temporal Transformer U-Net for Motion Artifact Reduction Using PAD (Pseudo All-Phase Clinical-Dataset) in Cardiac CT. IEEE Trans Med Imaging. 2023;42(12):3805-16.

13. Bunkhumpornpat C, Boonchieng E, Chouvatut V, Lipsky D. FLEX-SMOTE: Synthetic over-sampling technique that flexibly adjusts to different minority class distributions. Patterns (N Y). 2024;5(11):101073.

14. Lin TY, Goyal P, Girshick R, He K, Dollar P. Focal Loss for Dense Object Detection. IEEE Trans Pattern Anal Mach Intell. 2020;42(2):318-27.
