# Supplementary material for: Artificial intelligence risk stratification from dynamic digital subtraction angiography radiomics predicts pulmonary embolism and associates with clinical outcomes in deep vein thrombosis: A retrospective cohort study
Source: J Vasc Surg Venous Lymphat Disord. 2026 Feb 3;14(3):102450. doi: 10.1016/j.jvsv.2026.102450 (PMC12954298; doi:10.1016/j.jvsv.2026.102450)
Supplement: Supplementary Table 1 [file mmc2.docx]

**Supplementary Table I (online only): Cox Proportional-Hazards Regression Analysis for Predictors of Post-thrombotic Syndrome**

| **Variable** | **Univariate Analysis** | | **Multivariable Analysis** | |
| --- | --- | --- | --- | --- |
|  | **HR (95% CI)** | **P-value** | **HR (95% CI)** | **P-value** |
| Collateral Index  (per 0.1-unit increase)​ | 0.63 (0.55–0.72) | <0.001 | 0.62 (0.54–0.71)​​ | ​**​**<0.001 |
| Age (per 5-year increase) | 1.15 (1.02–1.30) | 0.023 | 1.08 (0.95–1.22) | 0.243 |
| Iliac Vein Involvement (Yes vs. No) | 2.45 (1.68–3.58) | <0.001 | 2.10 (1.42–3.11) | <0.001 |
| Active Malignancy  (Yes vs. No) | 1.82 (1.20–2.76) | 0.005 | 1.71 (1.12–2.62) | 0.013 |
| Abbreviations: CI, confidence interval; HR, hazard ratio. The multivariable model was adjusted for age, sex, thrombus location, and active malignancy status. | | | | |
