# Supplementary material for: Artificial intelligence risk stratification from dynamic digital subtraction angiography radiomics predicts pulmonary embolism and associates with clinical outcomes in deep vein thrombosis: A retrospective cohort study
Source: J Vasc Surg Venous Lymphat Disord. 2026 Feb 3;14(3):102450. doi: 10.1016/j.jvsv.2026.102450 (PMC12954298; doi:10.1016/j.jvsv.2026.102450)
Supplement: Supplementary Table 2 [file mmc3.docx]

**Supplementary Table II (online only): Ablation Studies on Model Architecture and Training Strategy**

| **Performance Metric** | **Proposed Hybrid Model** | **Baseline 3D-CNN**  **(Pre-trained + Fine-tuned)** | **P-value_1_ (Architecture)** | **Same Architecture Trained from Scratch** **(on DSA data alone)** | **P-value_2_**  **(Training Strategy)** |
| --- | --- | --- | --- | --- | --- |
| Feature Extraction Success Rate (%) | 98.7%​​ | 85.2% | ​​< 0.001 | 82.1% | <0.001 |
| AUC for PE Prediction (95% CI)​ | 0.88  (0.85–0.92)​​ | 0.84  (0.80–0.88) | 0.026 | 0.83(0.79–0.87) | 0.03 |
| Sensitivity (%)​ | 89.6% | 83.5% | 0.035 | 81.3% | 0.08 |
| Specificity (%)​ | 86.5% | 80.1% | 0.042 | 78.9% | 0.11 |
| F1-Score | 0.87 | 0.81 | 0.04 | 0.80 | 0.04 |
| Accuracy (%)​ | 87.2 | 81.8 | 0.09 | 80.5 | 0.09 |
| Average Training Time (Epochs, mean ± SD)​ | 45 ± 5 | 58 ± 8 | 0.005 | 75 ± 12 | <0.001 |
| Mean Training Loss (Focal Loss, mean ± SD)​ | 0.21 ± 0.03 | 0.26 ± 0.05 | 0.01 | 0.28 ± 0.07 | 0.01 |
| P-value₁: Comparison between the Proposed Hybrid Model and Baseline 3D-CNN (both pre-trained + fine-tuned), testing ​architecture superiority.  ​P-value₂: Comparison between the Proposed Hybrid Model (pre-trained + fine-tuned) and itself trained from scratch, testing ​training strategy efficacy.  ​Abbreviations: AUC, area under the curve; CI, confidence interval; PE, pulmonary embolism; SD, standard deviation. | | | | | |
