# Supplementary material for: Artificial intelligence risk stratification from dynamic digital subtraction angiography radiomics predicts pulmonary embolism and associates with clinical outcomes in deep vein thrombosis: A retrospective cohort study
Source: J Vasc Surg Venous Lymphat Disord. 2026 Feb 3;14(3):102450. doi: 10.1016/j.jvsv.2026.102450 (PMC12954298; doi:10.1016/j.jvsv.2026.102450)
Supplement: Supplementary Material [file mmc4.docx]

**
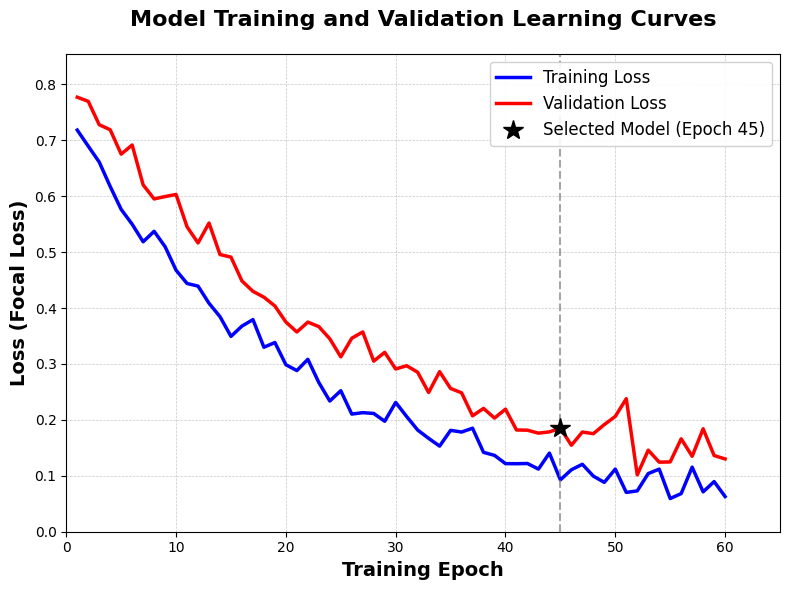
**

**Supplementary Fig 1. (online only).** **Model Training and validation learning curves.​​**

Training (blue) and validation (red) loss (Focal Loss) descend synchronously to a stable plateau, indicating effective generalization without overfitting. The vertical dashed line denotes the epoch (45) at which training was halted by early stopping (patience=20 epochs). The star marks the final model, selected from the epoch with the minimum validation loss prior to stopping.
